# Generation of primary human intestinal T cell transcriptomes reveals differential expression at genetic risk loci for immune-mediated disease

**DOI:** 10.1136/gutjnl-2013-306657

**Published:** 2014-05-05

**Authors:** Tim Raine, Jimmy Z Liu, Carl A Anderson, Miles Parkes, Arthur Kaser

**Affiliations:** 1 Department of Medicine, Addenbrooke's Hospital, University of Cambridge, Cambridge, UK; 2 Wellcome Trust Sanger Institute, Wellcome Trust Genome Campus, Hinxton, Cambridge, UK

**Keywords:** IBD, Intestinal T Cells, Genetic Polymorphisms, Coeliac Disease, Immune Response

## Abstract

**Objective:**

Genome-wide association studies (GWAS) have identified genetic variants within multiple risk loci as predisposing to intestinal inflammatory diseases, including Crohn's disease, ulcerative colitis and coeliac disease. Most risk variants affect regulation of transcription, but a critical challenge is to identify which genes and which cell types these variants affect. We aimed to characterise whole transcriptomes for each common T lymphocyte subset resident within the gut mucosa, and use these to infer biological insights and highlight candidate genes of interest within GWAS risk loci.

**Design:**

We isolated the four major intestinal T cell populations from pinch biopsies from healthy subjects and generated transcriptomes for each. We computationally integrated these transcriptomes with GWAS data from immune-related diseases.

**Results:**

Robust, high quality transcriptomic data were generated from 1 ng of RNA from precisely sorted cell subsets. Gene expression patterns clearly differentiated intestinal T cells from counterparts in peripheral blood and revealed distinct signalling pathways for each intestinal T cell subset. Intestinal-specific T cell transcripts were enriched in GWAS risk loci for Crohn's disease, ulcerative colitis and coeliac disease, but also specific extraintestinal immune-mediated diseases, allowing prediction of novel candidate genes.

**Conclusions:**

This is the first report of transcriptomes for minimally manipulated intestinal T lymphocyte subsets in humans. We have demonstrated that careful processing of mucosal biopsies allows the generation of transcriptomes from as few as 1000 highly purified cells with minimal interindividual variation. Bioinformatic integration of transcriptomic data with recent GWAS data identified specific candidate genes and cell types for inflammatory pathologies.

**Significance of this study**

**What is already known on this subject?**

- GWAS studies have identified large numbers of genetic risk loci associated with increased risk of developing intestinal inflammatory disorders, but most risk variants are non-coding and are believed to act through regulation of gene expression in a cell-type specific manner.
- Many IBD risk loci contain multiple ‘candidate genes’. For many the causal gene has not been defined and little is known about the cell types which may be most relevant for their functional effects.
- T cells resident within the intestinal mucosa represent the largest accumulation of T cells in the body and play a prominent role in regulation of mucosal inflammation.
- Intestinal T cells differ markedly from T cells in the peripheral blood in terms of surface marker expression. Little functional data is available for intestinal T cells and their gene expression patterns have never been characterised at a transcriptional level in healthy humans.

**What are the new findings?**

- We report the successful isolation and full transcriptional analysis of the four major T cell populations of the human intestinal mucosa.
- Precise bioinformatic analysis provides insight into human mucosal T cell specific gene expression patterns and signalling pathways.
- Overlaying expression and genetic data allows the identification of candidate genes and candidate cell types for explaining the functional impact of GWAS risk loci.

**How might it impact on clinical practice in the foreseeable future?**

- The identification of gut specific expression patterns and candidate genes at GWAS risk loci permits targeted therapeutic strategies for regulation of gut inflammation.
- The capacity to generate transcriptomic analysis of cell populations from biopsies offers a new approach to assess at a molecular level the cell-specific impact of therapeutic interventions.

## Introduction

Genetic mapping of susceptibility loci for common diseases is proceeding at pace^1–4^ but where many genes map to a risk locus the identity of the causal gene is difficult to discern. In most instances, risk associated genetic variants are predicted to affect transcriptional regulation.^5^ ^6^ Some of these effects may be identified through the generation of human genome-wide expression quantitative trait locus (eQTL) data sets.^7^ However, the cell-type specificity and modest magnitude of eQTL effects^7–9^ mandates isolation and purification of multiple cell types of interest from large numbers of donors. Consequently, for most primary human cell types such data remain unavailable. This is particularly true for cells located at sites of disease manifestation. Instead, investigators have hitherto had to infer insights from studies of murine tissue, transformed cell lines, human peripheral blood or *in silico* literature-mining in order to highlight candidate genes within a risk locus.^2^

The need to capitalise upon genetic data to gather functional insight is particularly felt in inflammatory diseases of the GI tract, where a number of high quality genome-wide association studies (GWAS) have been performed. Importantly, while a range of immunocytes are present in the GI mucosa and contribute to inflammatory homeostasis, T cells represent the dominant population.^10^ Intestinal T cells appear to be tissue resident, show minimal recirculation in the peripheral blood,^10^ ^11^ and exhibit fundamental differences to those found in other sites in terms of cell surface marker expression, activation pathways and putative function.^10^ ^12^ These cell populations therefore represent plausible candidates in which causal genetic variants might exert their effects. Practical limitations prevent the generation of an eQTL data set for human intestinal T cells due to difficulties accessing these populations in large numbers of subjects. As an alternative, transcriptomic data can provide a genome-wide assessment of population characteristics and allow unbiased identification of genes of functional relevance.^13^ In particular, those genes upregulated in intestinal T cells compared with a reference peripheral blood T cell population might be of particular importance for intestinal immune homeostasis and afford insight into the unique nature of intestinal T cell populations. We reasoned that testing of the overlap between these upregulated genes and GWAS risk loci for inflammatory disease would identify genes of importance for intestinal immune homeostasis potentially subject to transcriptional regulation modulated by disease-associated genetic variation. Further, that this would provide a novel approach to the identification of candidate risk genes.

Biological insight into human immunocytes has been dominated by studies in peripheral blood, and T cell populations in the healthy human intestine have never been characterised at a transcriptional level. Even in the better studied murine model system, where the profound differences of intestinal T cell differentiation and function compared with those found at other locales has been studied, there are only limited transcriptomic data for individual intestinal cell subsets,^14^ ^15^ including murine subpopulations without direct human equivalents.^16^

Intestinal T cells can be divided into two distinct populations: intraepithelial lymphocytes (IELs) reside interspersed among intestinal epithelial cells, and lamina propria lymphocytes (LPLs) are resident in the deeper stromal layer. In the present study our first aim was to generate transcriptomic profiles for the four most abundant T cell populations of the healthy human intestine (CD4 and CD8 IELs, and CD4 and CD8 LPLs), along with paired reference populations from peripheral blood. The transcriptional profile of each subset is here made available as a resource. Using strictly defined anatomical, physiological and pathological criteria, we have successfully minimised the interindividual variability that often confounds human studies, while an optimised experimental workflow, precise polychromatic flow cytometric sorting and robust computational analysis further increased data reliability. We next subjected these transcriptomes to *in silico* analysis to generate insight into activity within these cell populations through analysis of genes showing differential expression between intestinal and peripheral blood T cells. Finally, we sought to determine the enrichment of genes differentially overexpressed in these critical gut immune cell populations within risk loci identified by GWAS in a number of immune-mediated diseases, and align these to existing functional knowledge regarding genes at these loci.

## Methods

### Subject selection and sample collection

Six healthy non-smoking female subjects aged 33–52 years, taking no regular medications who were undergoing ileocolonoscopy for screening purposes, were recruited for biopsy collection. The terminal ileum (TI) was chosen for a number of reasons: it represents a reliable anatomical landmark, is the most common site of first presentation of Crohn's disease (CD),^17^ and is the site of highest density of luminal microbes in the small intestine.^18^ No appreciable abnormalities were noted macroscopically, nor were any histological abnormalities noted on routine biopsy analysis. Subjects received a standardised bowel preparation and underwent the procedure in the morning. Eight pinch biopsies were collected from the TI with visual avoidance of Peyer's patches as well as of previously sampled areas. Biopsies were collected into medium on ice and processed immediately, and 10 mL of peripheral blood was also collected into tubes containing 15 mg EDTA. Ethical approval was granted by the Cambridge National Health Service Research Ethics Committee, reference 01/418.

### Tissue processing

TI biopsies were suspended in calcium and magnesium free medium with 2 mM EDTA and 1 mM DTT and agitated for 30 min with three changes of media. At each stage, supernatants (containing the IEL fraction) were separated off using a 70 μm cell strainer. After the last media change, the remaining fragments were dissociated through incubation with 200 U mL^−1^ collagenase IV for 1 h, prior to washing through a 70 μm cell strainer. Peripheral blood mononuclear cells were separated by centrifugation over a Nycodenz density gradient. All isolation steps with the exception of enzymatic digestion were performed at 4° C. Cells were stained with an antibody cocktail comprising monoclonal antibodies (mAb) to CD19 (HIB19), CD326 (9C4), TCRαβ (IP26), CD62L (DREG-56), CD45RO (UCHL1), CD8a (HIT8a), CD4 (RPA-T4), CD3 (SK7) and CD8β (2ST8.5H7). After the final antibody wash step, 4′,6-diamidino-2-phenylindole was added to washed cells, to enable live/dead discrimination. A strict phenotypical definition was applied for sorting to avoid population contamination, as CD3^+^TCRαβ^+^ CD19^−^ CD326^−/lo^ 4′,6-diamidino-2-phenylindole^−^ CD62L^−^ CD45RO^+^, further subcharacterised as CD4^+^CD8α^−^ CD8β^−^ or CD4^−^ CD8α^+^ CD8β^+^. Cell separation was performed using a BD Facs Aria III cell sorter with cells sorted directly into chilled cell lysis and RNA stabilisation buffer (Buffer RLT, Qiagen) prior to storage at −80° C. Sort purity was verified to >99% prior to and after each sort. To minimise cellular perturbation, sorting was performed using a 100 µm nozzle at low sorting pressures using chilled, preservative-free PBS.

### RNA extraction and microarray preparation

Total RNA was isolated from sorted cells using RNeasy micro kits (Qiagen) for expression analysis using Human Gene ST 1.0 microarrays (Affymetrix). A number of specific laboratory and computational measures were undertaken to enable reliable transcriptomic analysis using just 1 ng of RNA from each cell population, as detailed in the online supplementary material. Genes showing differential expression were used for pathway analysis with Ingenuity Pathway Analysis (Ingenuity Systems) and enrichment significance determined by Fisher's exact test, and for protein-protein interaction modelling as detailed in the online supplementary material.

### GWAS interval enrichment analysis

Testing for enrichment of differentially expressed genes within genetic risk loci, as well as analysis of enrichment of transcription factor chromatin immunoprecipitation sites were performed as described in the online supplementary material (**Accession code.** GEO: microarray data, GSE49877).

## Results

### Generation of a high quality T cell transcriptome resource for human intestinal T cells

We extracted IELs and LPLs from pinch biopsies of the TI taken during ileocolonoscopy from six healthy, non-smoking female subjects aged 32–55 years taking no regular medications. Extensive measures were taken to minimise interindividual variation and *ex vivo* cellular perturbation (see online supplementary methods). Precisely defined CD4^+^ and CD8^+^ T effector memory (T_EM_) cells were purified from biopsies and paired peripheral blood samples according to a strict phenotypical definition, using polychromatic sorting (see methods). Peripheral blood T_EM_ cells may be identified as CD45RO^+^ CCR7^−^ and express variable levels of CD62L.^19^ However, we find CD45RO^+^ CCR7^−^ T cells in the gut do not express CD62L, and likewise gut CD45RO^+^ CD62L^−^ cells do not express CCR7 (20 and data not shown). For this reason, we used CD45RO^+^ CD62L^−^ to define T_EM_ cell populations. Since most gut T cells belong to the T_EM_ subset, in contrast to peripheral blood T cells, which predominantly bear a naïve or central memory phenotype,^21^ ^22^ this approach results in the identification of T_EM_ cells in the blood and gut with a closely matched cell surface activation phenotype and thus minimises biasing comparative analysis towards pathways associated with cellular activation and memory (see online supplementary figure S1A–F).

The use of fresh biopsy tissue from healthy individuals (rather than much larger tissue sections from the margins of diseased explants, where results are likely to be biased by the underlying pathology or perioperative factors including variation in ischaemic time and concomitant medication) resulted in cell yields in the 10^3^–10^4^ range. This necessitated RNA amplification with just 1 ng RNA of starting material from each population, which we achieved without the introduction of 3′ bias or loss of interarray signal array concordance that can occur with *in vitro* transcription based amplification technologies (see online supplementary figure S1G,H).^23^ In order to maximise reliable signal/noise discrimination and allow detection of low abundance transcripts that are often excluded from such analyses, without reduction in the statistical power to detect differentially expressed genes,^24^ we used a sophisticated analysis approach, based upon comparison of the signal from each of the probes in a given set to that from non-expressed control probes matched for GC-content. Pairwise analysis of gut T_EM_ cell populations with phenotypically matched cells from the peripheral blood of the same individual further increased statistical power.

### Human intestinal T cell transcriptomes reveal major differences from peripheral blood

**Figure 1:**
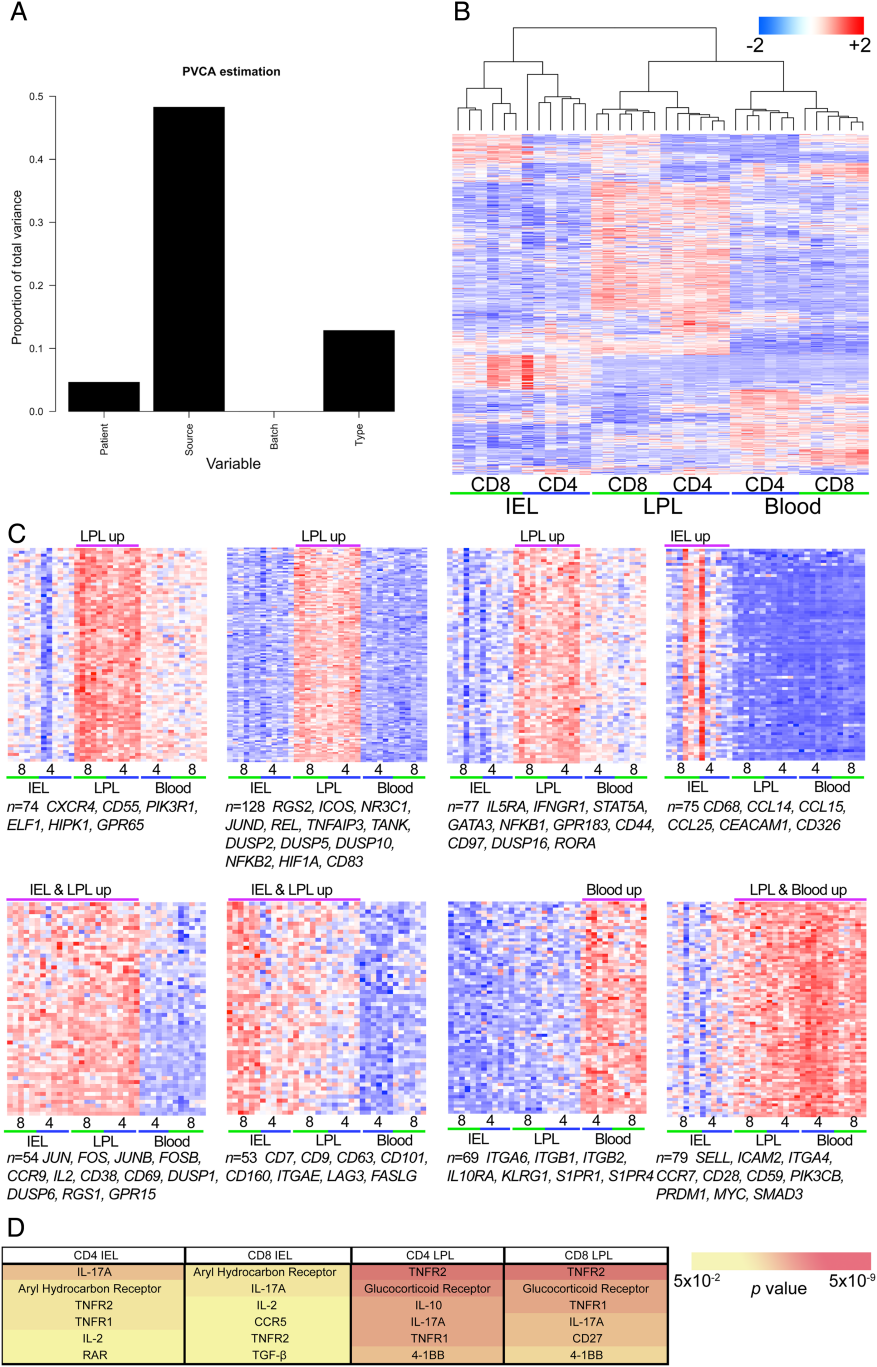
Analysis of human T_EM_ transcriptomes reveals compartment-specific signatures. (A) Effective removal of batch effects and minimisation of patient-of-origin variables within the expression microarrays. After initial background correction and normalisation, expression microarray data were subjected to correction for known potential batch effects within the data set. The effect size of known variables, including batch assignment, patient of origin, tissue source (IEL, LPL or blood) or cell type (CD4 or CD8) were then tested by PVCA which estimates the proportion of total variance within the data set that may be attributed to each variable. (B) Unsupervised hierarchical cluster analysis of transcriptomes from human gut and blood T_EM_ cells using the 500 transcripts showing the greatest variance. (C) *k*-means cluster analysis was performed on transcripts showing ≥1.4-fold change in mean expression values between at least one pair of T_EM_ cell populations and a within-sample coefficient of variation ≤0.5. Expression levels for genes in the 8 most discriminant clusters are shown, based upon maximisation of the between-group difference in centre-weighted expression values, with the number of transcripts in each cluster (*n*) indicated along with representative examples of transcripts from the cluster. Row mean-centred expression levels are shown, with red representing above average expression and blue representing below average expression (red-blue colour bar upper right). Array column order in C is preserved as determined by hierarchical clustering in B. (D) Top upregulated canonical immune signalling pathways based upon Ingenuity pathway analysis of transcripts upregulated in intestinal T_EM_ cell populations relative to paired peripheral blood T_EM_ populations. Each list of differentially expressed genes is assessed for the proportion mapping to relevant immune canonical signalling pathways, and the result compared with that observed in the entire data set to calculate significance. *p* values after correction for multiple testing are indicated by background colour (yellow-red colour bar bottom right). IEL, intraepithelial lymphocytes; LPL, lamina propria lymphocytes; PVCA, principal variance component analysis; T_EM_, T effector memory.

The expression data set is publically available (Gene Expression Omnibus accession number GSE49877 http://www.ncbi.nlm.nih.gov/geo). Within the total data set we detected 9468 gene transcripts that passed filtering criteria, of which 2868 (30.2%) showed differential expression between at least one pair of T_EM_ populations and 1712 (18.2%) showed differential expression between one or more gut populations and the paired peripheral blood T_EM_ population (≥1.4-fold change; adjusted p<0.05), already highlighting the fundamental differences in tissue-resident compared with peripheral blood T cells. Greater overlap of differentially expressed transcripts was observed between CD4^+^ and CD8^+^ T_EM_ cells from the same gut compartment than between LPLs and IELs (see online supplementary figure S2). Importantly, principal variance component analysis confirmed that the largest determinant of variance came from tissue source (LPLs vs blood vs IELs) with cell type (CD4^+^ vs CD8^+^) showing less contribution, and interindividual variation accounting for <5% of total variance (figure 1A), an observation borne out by unsupervised hierarchical clustering analysis (figure 1B). *k*-means clustering analysis identified transcripts showing similar expression patterns associated with IEL and/or LPL and blood T_EM_ populations (figure 1C).

### Intestinal T cells exhibit shared and compartment-specific differential expression of transcripts for T cell chemotaxis and activation

Transcripts showing differential expression in gut T_EM_ cells included several with little or no previously described role in T cell biology as well as many previously implicated from murine studies as involved in T cell trafficking, activation, regulation and co-stimulation. (figure 1C, see online supplementary figure 3A,B).

Transcripts significantly upregulated in all gut populations included integrin *ITGAE* (CD103, integrin α_E_), *CCR9*, *CCL20* and *RGS1* (regulator of G-protein signalling 1), all involved in homing of T cell to the gut,^25–28^ while *ITGA6* (integrin α_6_), *ITGB1* (integrin β_1_), *ITGB2* (integrin β_2_) and *CXCR3* were significantly downregulated across all gut populations. *CXCR3* is implicated in the trafficking of peripheral blood T_EM_ cells into inflamed bowel,^29^ ^30^ but does not appear to be highly expressed under homeostatic conditions by gut resident T_EM_ cells. *S1PR1* and *S1PR4* (sphingosine-1-phosphate receptors 1 and 4, respectively), regulators of T cell activation and lymphoid organ egress,^31^ were downregulated in gut T cells. Reflecting tight anatomical compartmentalisation, we noted a number of IEL-specific and LPL-specific adhesion molecule and chemokine associated transcripts, including *ICAM1*, *CD38*, *CD44*, *CD97*, *CCL3*, *CCR6* and *CXCR4* all upregulated in LPLs but not IELs, while *CEACAM1*, *CEACAM5*, *CCL14*, *CCL15* and *CCL25* were upregulated in IELs but not LPLs (figure 1C, and data not shown).

The critical effector T cell cytokine, interleukin 2 (IL-2), was upregulated across all gut populations. Although previous reports have shown high levels of activation in murine intestinal T cells compared with peripheral blood T cells of a largely naïve phenotype,^21^ our findings suggest a high basal level of activation under homeostatic conditions among human gut T cells even when compared with peripheral blood T_EM_ populations that were matched according to their surface activation marker phenotype. This seems to be particularly true of LPL T_EM_ cells (see online supplementary figure 3A). In keeping with this observation, we also found profound upregulation across all intestinal T_EM_ subsets of transcripts for *FOS*, *FOSB*, *JUN* and *JUNB*, components of the heterodimeric transcription factor activator protein 1 (AP-1). AP-1 signals downstream of the T cell receptor and multiple T cell co-stimulatory pathways drive IL-2 production, alongside transactivation of a variety of other inflammatory mediators. Other transcription factors synergise with AP-1 in driving IL-2 production, including *GABP*, *NFKB* and *EGR1*,^32^ all of which were significantly upregulated in CD4^+^ and CD8^+^ LPL T_EM_ cells (data not shown). This central role for nuclear AP-1 transcription factor activity, mediated through FOS and JUN was supported by evidence from protein-protein interaction modelling (see online supplementary figure S4), as well as from previous reports for murine IELs^21^ and LPLs.^14^ We also noted the expression of several transcripts associated with regulatory T cell activity, particularly among CD4^+^ LPLs, including *IL2RA*, *CTLA4*, *FOXP3* and *CD83*. Taken together, these findings point to a high degree of cellular activation with simultaneous tight regulatory control among human gut T cells under steady-state conditions, in keeping with previous reports in the mouse.^21^

### Intestinal T cell transcriptomes show evidence for T_H_17 and TNFR signalling alongside alternative co-stimulatory pathways

*BATF*, another AP-1 member, was significantly upregulated along with *IRF4* in CD4^+^ LPL T_EM_ cells; BATF/JUN/IRF4 complexes have been shown to bind diverse DNA elements and to be critical in directing T helper (T_H_) 17 cytokine production by acting as pioneer factors to open chromatin sites up to the binding of other T_H_17 transcription factors^33–35^ including AHR, RORA, STAT3, KDM6B and HIF1A,^35^ all of which also showed significant upregulation in LPL CD4^+^ T_EM_ cells. Other T_H_17 associated transcripts upregulated in CD4^+^ LPL T_EM_ cells included *IL1R1*, *IL22*, *IL23R* and *CCR6*. The role of T_H_17 cytokines for murine mucosal homeostasis is well recognised,^36^ and our findings are consistent with murine data showing small intestinal LP CD4^+^ T cells as the predominant source of IL17A under homeostatic conditions.^37^ It is also interesting to note the prominent role for T_H_17 pathway members highlighted by genetic studies of susceptibility to IBD.^38^

The importance of IL-17A signalling in intestinal T cells was supported by pathway analysis, which also showed evidence for signalling through both receptors for tumour necrosis factor α, TNFR1 and TNFR2, as well as multiple alternative pathways of T cell activation and regulation, including the glucocorticoid receptor (NR3C1), CD27 and 4-1BB (figure 1D). Protein-protein interaction modelling reinforced these findings, with evidence of NR3C1 signalling in LPL T cell networks, as well as NF-κB signalling (see online supplementary figure S4), consistent with TNFR signalling as suggested by pathway analysis. Indeed, multiple NF-κB pathway members were all upregulated in CD4^+^ and CD8^+^ LPL T_EM_ cells, including *NFKB1*, *NFKB2*, *REL* and *RELB*, as well as their associated regulatory and target genes, including *NFKBIA* (IκBα), *NFKBID* (IκBδ), *NFKBIE* (IκBε) and *NFKBIZ* (IκBζ).

### Risk loci for GI inflammatory pathologies are selectively enriched for transcripts upregulated in gut T cells

Genes differentially expressed in gut T_EM_ cell populations relative to peripheral blood T_EM_ cell populations may be predicted to be important for gut immune homeostasis. Perturbations of this homeostasis are implicated in a number of inflammatory diseases in humans, affecting the intestine itself and other organ systems.^39–41^ We reasoned that GWAS of intestinal inflammatory diseases might identify genetic loci containing genes important for intestinal immune homeostasis, and hence be enriched for genes showing differential expression in intestinal T cells under steady-state conditions.

**Figure 2:**
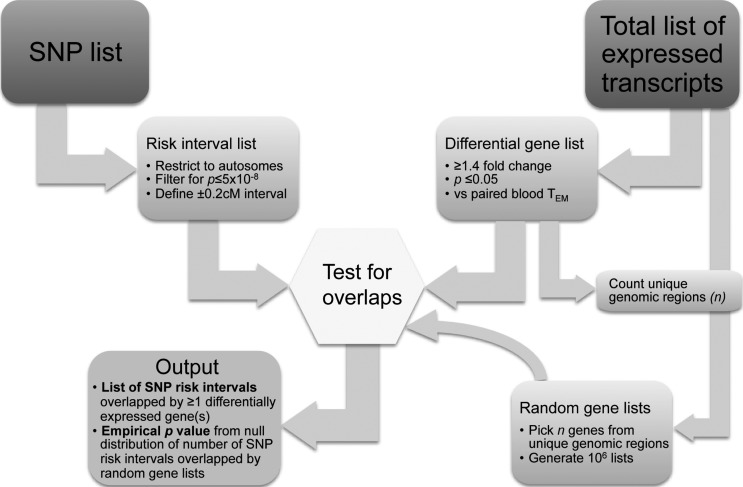
Overview of the algorithm for testing for association between focal SNP lists from GWAS studies and lists of differentially expressed genes. Lists of focal SNPs were filtered for genome wide significance and autosome mapping, and assigned a genomic interval extending 0.2 cM either side. The fraction of SNPs with intervals overlapping one or more differentially expressed transcripts was then calculated. The significance of this overlap was calculated, based upon repeated testing of the overlap of a random selection of transcripts from the list of all transcripts expressed in the data set, regardless of relative expression. In each iteration of the random sampling algorithm, the number of transcripts selected corresponded to the total number of unique genomic regions represented in the original differential gene expression list. GWAS, genome-wide association studies; SNP, single nucleotide polymorphism; T_EM_, T effector memory.

To this end, we defined a genomic interval extending 0.2 centiMorgan (cM) either side of the ‘focal’ single nucleotide polymorphism (focal SNP—ie strongest association signal) for each confirmed GWAS susceptibility locus for a range of common diseases and traits. We then assessed the frequency with which these intervals contained a transcript which we found to be upregulated or downregulated in any of the four gut T_EM_ populations relative to their paired peripheral blood T_EM_ population. We compared the degree of overlap observed with background distributions generated by repeat testing of equivalent numbers of transcripts selected at random from the total data set of T_EM_ expressed transcripts, regardless of differential expression (figure 2).

**Figure 3:**
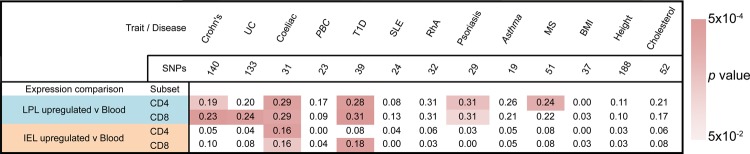
Risk loci for intestinal and non-intestinal inflammatory pathologies are enriched for genes upregulated in gut T_EM_ cell populations. Genetic risk loci associated with a range of diseases and traits, as indicated, were tested for overlap with transcripts showing upregulated expression in specific intestinal T_EM_ cell populations relative to paired peripheral blood T_EM_ cell populations, according to the algorithm illustrated in figure 2. The proportion of risk intervals containing one or more differentially expressed genes within a window extending 0.2 cM either side of the lead SNP, is shown for each trait/gene list combination, with background colouring indicating the significance of the observation, as per the legend. The total number of risk intervals tested for each condition, after filtering for genome wide significance and overlapping intervals, is shown at the head of each column; citations for lists of SNPs used are given in Methods. SNP, single nucleotide polymorphism; T_EM_, T effector memory.

We first tested three common GI inflammatory diseases for which high quality GWAS data have been generated and which demonstrate confirmed association with multiple independent risk loci: CD, ulcerative colitis (UC) and coeliac disease (CeD). For all three diseases, we noted marked convergence between genetic risk loci and transcripts upregulated in LPL T_EM_ cells relative to peripheral blood, reaching statistical significance in all instances apart from CD4^+^ LPL T_EM_ cells in UC (figure 3). In keeping with evidence for IELs and LPLs playing a key role in the pathogenesis of CeD^42^ ^43^ we also found significant enrichment at CeD risk loci for transcripts upregulated in IEL T_EM_ cells. More significant enrichment observed with CD and CeD compared with UC risk loci may reflect the colonic focus of UC, whereas in our study the T_EM_ cells were extracted from small bowel biopsies.

No significant enrichment for any of the three diseases was noted for genes downregulated in any gut population (ie upregulated in peripheral blood T_EM_ relative to gut T_EM_, online supplementary figure S5), nor did we detect any significant enrichment for differentially expressed genes with risk loci for non-inflammatory polygenic traits tested: body mass index, height or blood cholesterol levels (figure 3).

**Figure 4:**
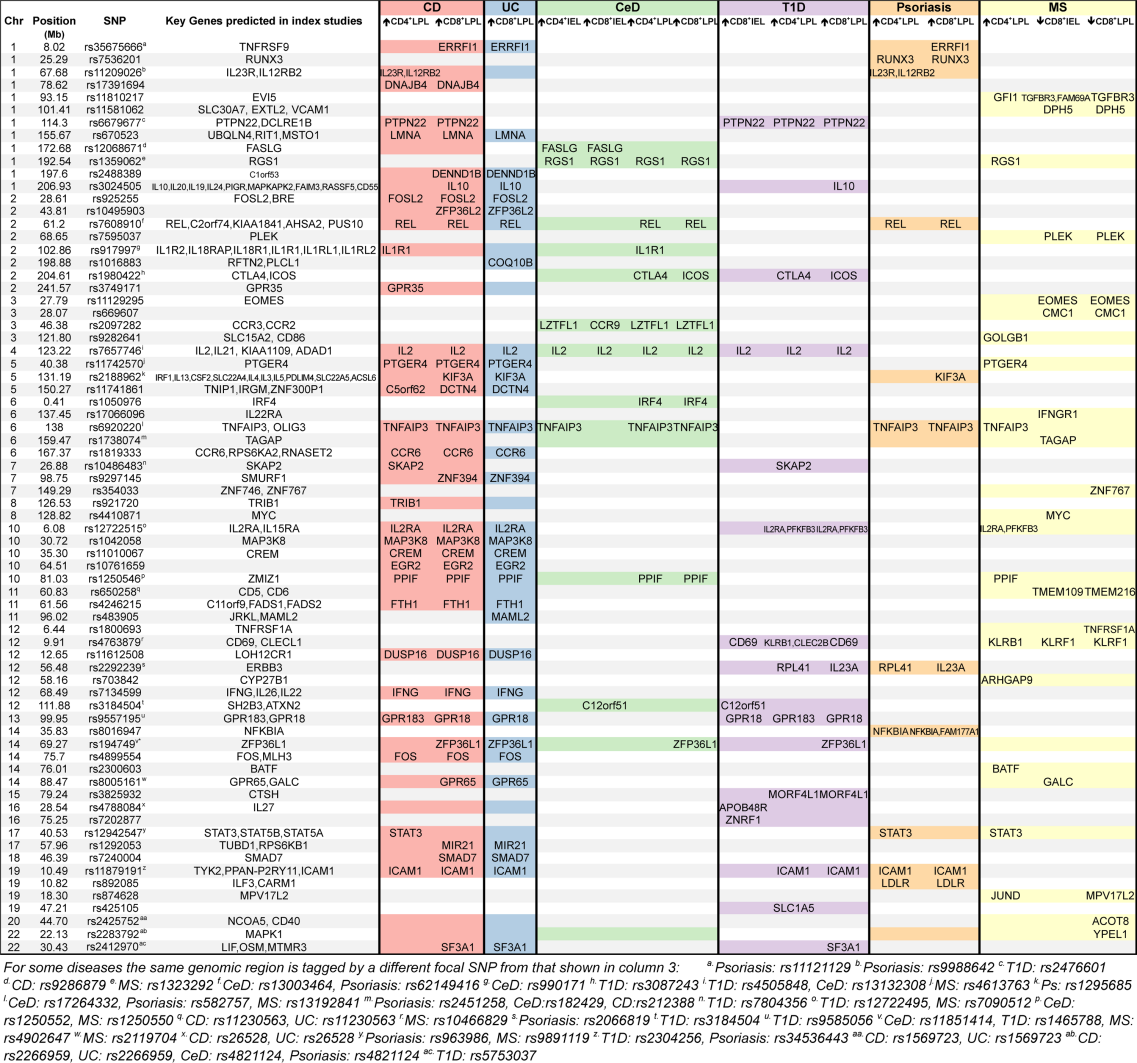
Annotation of disease-associated SNPs with genes showing differential expression in intestinal T_EM_ populations. For each of disease SNP-differential gene list combinations for which the degree of overlap reaches statistical significance (as shown in figure 3), those risk loci (encompassing a 0.2 cM window either side of the focal SNP) that contain a gene differentially expressed in a gut T_EM_ cell population are shown. Also indicated are the positional candidate genes identified as of interest in the original papers reporting genetic association. Where a risk locus is associated with a specific disease, the intersection between the locus (rows) and disease (columns) is shaded; where this is associated with a differentially expressed gene, the gene name(s) are indicated in the subcolumn for the appropriate T_EM_ population. Note that where there is an association between a risk locus and a disease, but no differentially expressed gene at that risk locus in a given T_EM_ subpopulation, the corresponding cell is shaded, but left empty. Note also that for risk loci shared between different diseases, the focal SNP may vary between diseases as indicated in the figure footnote. BMI, body mass index; IEL, intraepithelial lymphocytes; LPL, lamina propria lymphocytes; MS, multiple sclerosis; PBC, primary biliary cirrhosis; RhA, rheumatoid arthritis; SLE, systemic lupus erythematosus; SNP, single nucleotide polymorphism; T1D, Type 1 diabetes; T_EM_, T effector memory.

We next tested a number of diseases where the inflammatory pathology occurs outside of the intestine. We did not detect any significant enrichment for asthma, primary biliary cirrhosis, rheumatoid arthritis or systemic lupus erythematosus. However, there was a highly significant overlap between Type 1 diabetes (T1D)-associated loci and genes upregulated in CD4^+^ and CD8^+^ LPLs and CD8^+^ IELs. Likewise, we found significant overlap for psoriasis risk loci and genes upregulated in both LPL T_EM_ populations; for multiple sclerosis (MS), risk loci showed significant overlap with genes upregulated in CD4^+^ LPL T_EM_ cells, but also with genes downregulated in CD8^+^ IEL and LPL T_EM_ cell populations. Several of these immune diseases share multiple genetic susceptibility loci. However, the enrichment around SNPs associated with extraintestinal pathology was not solely restricted to those genetic loci shared with GI inflammatory diseases (figure 4).

UC and CD represent two related forms of IBD, with considerable overlap of genetic risk loci.^2^ In order to test for the signature of a common transcription factor whose activity might link gut T cell expressed genes with risk loci for IBD, we employed chromatin immunoprecipitation enrichment analysis, a computational approach based on the identification of clusters of upregulated and downregulated genes sharing common transcription factor binding sites in their promoter regions, as identified by chromatin immunoprecipitation assays.^44^ We identified HNF4a as a potential key transcription factor associated with patterns of gene expression seen in CD4^+^ and CD8^+^ LPL T_EM_ cells. Importantly, binding sites for HNF4a were significantly enriched at IBD associated SNPs that lay close by an LPL T_EM_ upregulated gene, but not at IBD associated SNPs that do not map to upregulated genes (*p*=0.01, see online supplementary table S1). Deletion of *Hnf4a* in murine intestinal epithelial cells results in spontaneous colitis,^45^ and *HNF4a* is a candidate gene at the UC risk locus tagged by rs6017342^2^; however, a role for HNF4a in T cell homeostasis remains unexplored.

## Discussion

Using a range of experimental and bioinformatic procedures to overcome the barriers associated with the study of primary human GI T cells under homeostatic conditions, we report here unbiased expression microarray data for minimally manipulated T cells from the small intestine of tightly matched, healthy controls. We demonstrate that this approach allows the identification of genes upregulated in specific gut T cell subsets and that these genes cluster non-randomly around risk loci for inflammatory pathologies, thus providing an alternative novel approach to candidate gene identification at GWAS risk loci.

The only previously reported human intestinal T cell transcriptional studies used prolonged *in vitro* culture and expansion of clones derived from atypical IEL T cells associated with refractory CeD.^46^ ^47^ Here, we used material from just eight ileal biopsies, allowing study of physiologically relevant tissue from healthy control individuals rather than dependence upon diseased explants. Low cell yields associated with the use of biopsies and high stringency cell sorting, necessitated working with 1 ng starting RNA, and hence required rigorous laboratory and bioinformatic procedures to generate reliable microarray data (see online supplementary figure. S1G,H). Furthermore, through these procedures we were able to minimise interindividual variation to a remarkable extent (figure 1A) and to demonstrate statistically significant differences in gene expression using just six donors. Notably a recent report of tissue-specific patterns of gene expression for primary human dendritic cells, isolated from four skin donors and six unpaired blood donors, gained meaningful insight despite comparatively small sample sizes.^48^ Likewise, other investigators have performed insightful transcriptomic analysis of human intestinal dendritic cell subsets using unpaired samples from three to five individuals per cell type with comparison to peripheral blood and skin biopsies from different individuals.^49^ The pairwise comparison of samples from the same individual that we were able to make in our study further underpins the validity of our analysis.

We went to extensive lengths to minimise cellular perturbation by keeping the isolation process brief and working at 4° C wherever possible. Cell sorting, in particular, can induce cellular stress. For this reason we used chilled, preservative-free flow buffers at low pressures with a large nozzle size, and sorted cells directly into a lysis and RNA stabilisation buffer to minimise the risk of effects on RNA transcription. Exposure of peripheral blood mononuclear cells to the conditions used for IEL and LPL isolation prior to cell sorting did not lead to significant detectable changes in T_EM_ gene expression, reducing the possibility of transcriptomic differences arising as an artefact of cell isolation (see online supplementary figure S6).

The gut transcriptomes generated in the current study for the four most abundant human intestinal T cell populations now allow interrogation of gene expression in these critical populations in man. It is notable that 25% of known IBD risk loci contain a gene showing differential upregulation within an LPL T_EM_ cell population under homeostatic conditions, while 35% of CeD loci contain a gene upregulated in LPL or IEL T_EM_ cells. Although we chose a window size of 0.2 cM either side of the focal SNP, in line with previous studies of the *cis*-acting regulatory effects of genetic variants,^8^ similar significant enrichment at risk loci was seen when using smaller window sizes of 0.1 cM (data not shown). Our choice of threshold for genes showing differential expression of ≥1.4-fold change is based upon estimates of the threshold for reliability of expression microarrays from quantitative PCR validation studies^50^ and is consistent with the approach taken in recent T cell transcriptomic studies.^51^ Reassuringly, repeating our analysis of GWAS risk loci for enrichment in genes showing ≥2-fold change revealed minimal differences in the enrichments observed compared with those we had observed using genes showing ≥1.4-fold change (data not shown).

This enrichment points to the importance of genes involved in intestinal T cell homeostasis in predisposition to gut inflammation and hence to the primacy of these cells and genes in disease pathogenesis. That GWAS risk loci might also be enriched for genes showing upregulation in intestinal immunocytes under conditions of already established intestinal inflammation is not investigated in the present study, but merits further exploration. Nonetheless, there is a strong rationale to study genetic relationships in cells from healthy individuals,^52^ in keeping with a variety of important studies in the field.^53–55^ The use of tissue from healthy individuals represents an opportunity to study the relevance of GWAS loci in biologically relevant cell types and represents a logical extension to prior attempts to understand GWAS data in the context of immortalised cell lines^56^ or *ex vivo* differentiated tissue.^57^ Ultimately, identifying causal genes and linking their altered expression to GWAS risk SNPs requires a plausible biological mechanism or demonstration of allele-specific expression, or both,^53^ tasks which are technically challenging even when working with primary human cell types that can be sampled with relative ease, such as peripheral blood. The tissue-specificity of intestinal transcriptomic signatures we report serves as an important reminder of the fundamental limitations in attempts to infer biological insight from such readily accessible peripheral blood cell types.

In many instances, the gene-SNP overlaps we observe implicate an already proposed candidate gene, lending support to existing analyses, as well as highlighting the potential importance of a specific population of gut immune cells (figure 4). For example, the IBD associated risk variant rs1819333 on chromosome 6 lies over 160 kB upstream of *CCR6*, a candidate gene previously implicated from *in silico* analysis; our finding of differential upregulation of transcripts for CCR6 in LPL T_EM_ lends weight to this case. Likewise, rs11742570 lies almost 270 kB upstream of the LPL T_EM_ cell upregulated gene *PTGER4*, which encodes a prostaglandin E receptor shown to regulate T cell activation and T_H_17 signalling^58^ and which has been identified as a candidate IBD risk gene through *in silico* analysis.^2^ In contrast, in a gene-dense region of chromosome 2 with risk variants associated with IBD, CeD and psoriasis, previous *in silico* analyses have suggested multiple alternative candidate genes^2^ ^4^ ^59^; we find that at this locus the canonical NF-κB transcription factor *REL* is strongly upregulated in LPL T_EM_ cell populations compared with peripheral blood T_EM_ cells.

In other instances our approach offers new leads in loci devoid of obvious candidate genes, or, as important, highlights alternatives to genes previously proposed (figure 4). For instance, rs35675666 shows significant association with IBD risk and lies close to multiple genes, including *TNFRSF9*, which was previously highlighted as a likely positional candidate gene by *in silico* analysis.^2^ In our study, an alternative gene within this region, *ERRFI1*, showed high levels of expression in CD8^+^ LPL T_EM_ cells. *ERRFI1* encodes a cytoplasmic protein mediating negative inhibition of signalling through the family of epidermal growth factor receptors, which share a common signal transduction pathway through ERK-MAPK with the T cell receptor. Importantly, epidermal growth factor-receptor mediated signalling has recently been reported to modulate intestinal T cell regulation in a murine colitis model,^60^ and ERRFI1 is also highly expressed in murine LPL T cells, but not splenic T cells.^14^ We also noted high LPL T_EM_ transcription of another regulator of MAPK signalling, *DUSP16*; the IBD-associated SNP rs11612508 lies within a *DUSP16* intron, but the candidate gene previously reported at this locus is a gene of unknown function, *LOH12CR1*, based upon eQTL effects reported in adipose tissue.^2^ ^61^

Other examples of informative gene-SNP overlaps we observed include rs17391694, which marks a region on chromosome 1 associated with CD risk, and lies 140 kB downstream of *DNAJB4*, which we show to be highly expressed in LPL T_EM_ cells. *DNAJB4* is a HSP-40 family member, only recently described and barely studied in the literature; consequently, *in silico* analysis did not detect any candidate genes within the rs17391694 risk locus.^2^ Of multiple other similar examples for IBD, we note our LPL T_EM_ transcription based identification of *DENNDB1*, a guanine nucleotide exchange factor upregulated in memory T cells,^62^ as a candidate gene for rs2488389 (*C1orf53* previously reported based upon *in silico* analysis); *ZFP36L2*, a putative regulator of thymocyte differentiation,^63^ for rs10495903 (no previous candidate gene reported); *ZNF394*, a barely studied gene intriguingly reported to regulate AP-1 signalling,^64^ for rs9297145 (*SMURF1* previously reported based upon lymphoblastoid cell line eQTL analysis^65^); *EGR2*, a recently reported regulator of T_H_17 effector function^66^ for rs10761659 (no previous candidate gene reported); and *PPIF*, a recently reported regulator of apoptosis as well as chemotaxis^67^ for rs1250546 (no previous candidate gene reported).

Normal development and function of murine, and by inference human, intestinal T cells is critically dependent upon microbial cues,^18^ suggesting that many of the pathways we have identified will be driven by interactions with the gut microflora. Indeed, we note significant overlap between transcripts upregulated in CD4^+^ LPL T_EM_ cells and previous reports of the transcriptional changes induced in murine CD4^+^ LPL after colonisation of germ-free mice.^14^ Considerable evidence now supports the observation that the intestinal microbiota modulates local inflammation but also systemic immune-mediated pathologies.^41^ Indeed, T1D and MS exhibit evidence for disease modification by the gut microbiota in animal models and human studies.^39^ ^40^ ^68^ In this context, our observation that risk loci for T1D, MS and psoriasis are significantly enriched for genes overexpressed in specific populations of gut T cells may potentially offer novel insight into mechanisms of host-environment interactions in these common diseases.

In summary, we report the first transcriptomes for the four major human gut T cell populations and highlight substantial differences from peripheral blood T cells, suggesting novel, potentially gut-specific targets for T cell modulation. Using biopsies from a modest number of healthy study subjects allied to careful isolation and sophisticated bioinformatic analysis techniques, we show that the human intestinal T cell compartment can be made accessible for transcriptomic studies and may provide useful insight into genetic loci associated with inflammatory pathologies. The opportunity now arises to interrogate other human tissue resident cell transcriptomes in a variety of disease states, including those highlighted in the current study.

## Supplementary Material

Web supplement

Web figures
