# Supplementary material for: Generation of primary human intestinal T cell transcriptomes reveals differential expression at genetic risk loci for immune-mediated disease
Source: Gut. 2014 May 5;64(2):250–9. doi: 10.1136/gutjnl-2013-306657 (PMC4316924; doi:10.1136/gutjnl-2013-306657)
Supplement: Web supplement [file gutjnl-2013-306657-s1.pdf]

Supplemental online material for Raine *et. al*

SUPPLEMENTARY METHODS

RNA extraction and microarray preparation. RNA extraction was performed using RNeasy micro kits (Qiagen) in an automated manner on the QIAcube platform. Extracted RNA was subject to quantification and quality assurance using an Agilent 2100 Bioanalyzer and samples checked for a RNA Integrity Value (RIN) >7 prior to further analysis. Degradation of RNA from the 5' end may introduce bias which is further compounded by *in vitro* transcription based amplification technologies that preferentially amplify the 3' end of the RNA molecule[1]. However, careful RNA processing and the use the Ovation Pico system (NuGen Technologies) for single primer isothermal amplification resulted in no appreciable 3' bias (Supplemental Fig. 1g) with strong inter-array signal intensity concordance (Supplemental Fig. 1h) using 1ng starting material from all samples. Amplified RNA was then biotinylated using the Encore Biotin Module (NuGen) prior to hybridization to Human Gene ST 1.0 microarrays (Affymetrix) and reading using an Affymetrix GeneChip Scanner 3000 7G with data extraction using the Affymetrix Expression Console 3.2.3.1515 software. All arrays were required to pass initial quality control metrics including a positive versus negative area under the curve (AUC) ≥0.8.

Microarray pre-processing. Initial microarray data pre-processing was performed using Affymetrix Power tools (APT 1.15.0, Affymetrix) including robust multichip average (RMA) normalization. All steps from RNA extraction to array hybridization were performed in batches to which samples were randomly

assigned, with subsequent correction for any batch effects using the methods of Johnson *et al*[2]. Removal of batch effects was confirmed and effect size of known variables measured using principle variance component analysis (PVCA) according to the methods of Li. *et al*.[3] (Fig. 1a).

A common problem with expression microarray data is discrimination of signals from background noise, particularly for low abundance transcripts. Although noise will be constant between arrays and hence false detection of a significant difference between biological groups is unlikely, inclusion of signals from these probes in downstream analysis will incur a penalty in correction for multiple testing and thus lead to type II errors. Approaches using array designs based upon pairing each perfect match (PM) oligomeric probe with a deliberately mismatched probe (MM) altered at a single base enable estimation of signal specificity based upon PM/MM signal ratio but reduce the total number of transcripts that can be interrogated for any given number of probes.

PM-only design chips such as the Affymetrix Gene ST used in this study need an alternative approach to transcript presence/absence calling. Discarding results from probesets in an arbitrary bottom fraction of expression values[4] does not allow the discrimination of consistent biological differences in expression of low abundance transcripts. We used an approach based upon the APT Detection Above BackGround (DABG) algorithm, whereby every probeset is assigned a *p* value representing the probability of the signal representing background noise, based upon the comparison of the signal from each of the probes in a given probeset and from non-expressed control probes matched for GC-content[5]. We

defined a transcript as detected on an array if ≥50% of probesets mapped to a given transcript ID had a DABG *p* value ≤0.01; likewise, we defined a transcript as present within the total dataset if ≥50% of arrays for any single given cell type showed detection of the transcript according to these criteria (i.e. DABG *p* values ≤0.01 for ≥50% of probesets mapped to a single transcript ID in ≥3 arrays for any given T_{EM} cell population). Modelling of alternative thresholds showed that this combination of stringent DABG *p* value but relaxed probeset frequency resulted in the greatest power to detect differentially expressed transcripts in downstream analysis.

DABG filtering, removal of control or cross-hybridizing probesets and removal of probesets that were not mapped to autosomes or to chromosome X, reduced an initial dataset of 33,321 transcript IDs to 14,315. Additional filtering was performed to remove probesets mapped to transcripts that did not correspond to a confirmed RNA sequence in the National Centre for Biotechnology Information (NCBI) Reference Sequence (RefSeq) database (accession codes beginning 'NM' and 'NR'). This further reduced the total dataset to 9,468 transcript IDs.

Differential expression analysis. Transcript IDs passing pre-processing and filtering were taken forwards for differential gene expression using the R 'limma' software (Linear Models for Microarray Data; version 3.16.7), with weightings applied for array quality[6] and correction for multiple testing according to the methods of Benjamini and Hochberg[7]. Data were analysed pairwise, with each gut T_{EM} population paired to a corresponding peripheral blood T_{EM} population

taken from the same individual ($CD4^+$ T_{EM} IELs and $CD4^+$ T_{EM} LPLs paired to $CD4^+$ T_{EM} blood cells; $CD8^+$ T_{EM} IELs and $CD8^+$ T_{EM} LPLs paired to $CD8^+$ T_{EM} blood cells). Lists of differentially expressed genes were used for pathway analysis using Ingenuity Pathway Analysis (IPA, Ingenuity Systems) with enrichment significance determined by Fisher's Exact test.

Protein-protein interaction. Protein-protein interaction networks were built for lists of differentially expressed genes. Since not all proteins within a given pathway might need to be upregulated at the mRNA level in order to see enhanced signalling through that pathway, we first identified all protein binding partners for products of upregulated transcripts, then built an interaction network for all protein-protein interactions in this extended list. Protein-protein interactions were identified using iRefIndex, an expert curated database (http://irefindex.org; version 12.0)[8] and the results filtered to show only interaction data from *in vitro* studies of human proteins (i.e. *in silico* predictions and data from other species removed). Interactions with CFTR and UBC were removed since these are promiscuous in their associations, and then proteins left unlinked to the main network were also removed.

Results were plotted using Cytoscape software (http://www.cytoscape.org; version 3.0). Each protein reflected by a single node, with node prominence (size and shading) set to reflect betweeness centrality, a measure of the importance of each node to overall network connectivity, based upon the number of shortest paths between all other nodes in the network passing through the node itself[9]. Each protein-protein interaction is represented by an edge, with edge

prominence (size and transparency) set to reflect edge betweeness, a measure of the importance of each protein-protein interaction to overall network connectivity, based upon the number of shortest paths in the network passing through the edge itself, normalized to the total number of edges in the nodes that the edge belongs to (this normalization prevents proteins for which more interaction data has been reported from dominating the network view)[9]. Distance and positioning of each node within the network was determined according to a force-directed paradigm where nodes mutually repel unless drawn together by edges, according to the 'yFiles organic' algorithm (yWorks GmbH), with slight modifications only to ensure clarity of view.

GWAS interval enrichment analysis. Testing for enrichment of differentially expressed genes within genetic risk loci was performed using script written in Python (http://www.python.org; version 2.7.2). Lists of genetic risk loci associated with specific traits were drawn from definitive studies in the published literature (CD[10], UC[10], CeD[11], Psoriasis[12]), from the Immunobase Consortium (T1D, http://www.immunobase.org [accessed 1/4/13]) or from the National Institutes of Health (NIH) GWAS catalogue (http://www.genome.gov/gwastudies [accessed 1/4/13]). SNP lists were filtered for genome-wide significance (*p* value $\leq 5x10^{-8}$) and restricted to autosomes only since not all GWAS studies include chromosome X. Risk intervals based upon genetic distance were defined 0.2 cM (estimated using Phase I data from the 1000 Genomes project: http://www.1000genomes.org) either side of the focal SNP. Where multiple SNPs fell within overlapping recombination windows, the SNP with the less significant *p* value was removed from testing.

Lists of differentially expressed genes (DEGs) for the four gut T_{EM} populations were reanalysed according to the methods already described, with additional filtering prior to Limma analysis to restrict expression data to autosomes only (Limma analysis performed on 9,144 transcripts, rather than 9,468). Transcripts showing ≥1.4 fold differential expression with *p* value ≤0.05 after adjustment for multiple testing were used for analysis. We then counted the number of risk intervals that overlap with at least one DEG, defined here as the interval between the first start and last stop site of the transcript.

To assess the statistical significance of this result, we calculated the number of risk intervals that overlap with sets of transcripts picked at random from the total list of 9,144 expressed autosomal transcripts. This generates a null distribution of overlap counts from which the empirical *p*-value of the observed result can be obtained. The number of transcripts to sample was set initially as the same total number of DEGs that was observed in the DEG list for the comparison under test. However, since gene expression may occur non-randomly with respect to genomic position, the significance of the observed overlap may be biased by overrepresentation of genomic regions selected at random from DEG clusters. To avoid this, we sampled a number of transcripts equivalent to the number of DEGs representing unique genetic locations, as determined by removing from the list of DEGs any transcript that overlaps with an interval 0.2 cM either side of the midpoint of another DEG transcription interval. Likewise, during the random sampling, each new transcript picked was rejected if it fell within a 0.2 cM window around the midpoint of any transcript already selected. We assessed the degree of overlap of this random sample of

transcripts with the genetic risk intervals for the trait under study. This random sampling was iterated 10^6 times to generate a null distribution of the number of overlapping loci between genes present in our total dataset and the genetic risk loci. The empirical *p*-value is then the number of times the simulated number of overlapping risk loci exceed the observed number of overlaps divided by the number of iterations. In this way, we were able to assess the statistical significance of the observed overlap between any given list of genetic risk loci and any list of differentially expressed genes, without biasing for the total number of SNPs or genes in either list.

Chromatin Immunoprecipitation Enrichment analysis. To identify potentially active transcription factors (TFs) within the cell populations under study, lists of upregulated genes were analysed using the X2K algorithm of Chen *et al.*[13], based upon identification of genes known to coimmunoprecipitate with specific TFs in an expert curated database of published human chromatin immunoprecipitation sequencing (ChIP-Seq) data. TFs with ChIP-Seq binding sites occurring at a significantly higher frequency (after correction for multiple testing[7]) within the list of differentially expressed genes than in the master database represent putative active regulators within that cell population.

In order to identify potential TF binding sites modified by SNPs associated with IBD, focal IBD-associated SNPs as well as all SNPs in tight linkage disequilibrium ($r^2 \geq 0.9$) with the focal SNP, were identified using SNAP, an online SNP proxy search tool[14]. All SNPs were then analysed for the presence of known TF

binding sites based upon ChIP-sequencing data using an online, expert curated database[15].

We wanted to test whether any of the TFs identified as putative drivers of differential gene expression in LPL $CD4^+$ and $CD8^+$ T_{EM} cells might explain the association seen between IBD risk loci and genes over-expressed in these cell populations. We reasoned that such a TF might be expected to have multiple binding sites associated with those IBD risk SNPs that were in turn associated with a gut overexpressed gene, but significantly fewer binding sites associated with those IBD risk SNPs showing no such association.

To this end, we divided SNP lists into those where the focal SNP marked a risk locus containing a gene upregulated in LPL $CD4^+$ or $CD8^+$ T_{EM} cells, and those where no such overlap was observed. We then noted the distribution between these two lists of binding sites for those TFs identified as active in each cell population. For those TFs showing a minimum of five binding sites in each list, we calculated the probability of the null hypothesis of random distribution between the two lists by χ^2 testing, with adjustment for multiple testing[7].

REFERENCES FOR SUPPLEMENTARY METHODS

1 Clement-Ziza M, Gentien D, Lyonnet S, Thiery JP, Besmond C, Decraene C. Evaluation of methods for amplification of picogram amounts of total RNA for whole genome expression profiling. *BMC Genomics* 2009;**10**:246.

2 Johnson WE, Li C, Rabinovic A. Adjusting batch effects in microarray expression data using empirical Bayes methods. *Biostatistics* 2007;**8**:118-27.

3 Li JB, P.R.; Chu, T.-M.; Wolfinger, R.D. Principal variance components analysis: Estimating batch effects in microarray gene expression data. In: Scherer A, ed. Batch Effect and Experimental Noise in Microarray Studies: Sources and Solution. West Sussex, United Kingdon: John Wiley & Sons, 2009.

4 Shah SH, Pallas JA. Identifying differential exon splicing using linear models and correlation coefficients. *BMC Bioinformatics* 2009;**10**:26.

5 Lockstone HE. Exon array data analysis using Affymetrix power tools and R statistical software. *Brief Bioinform* 2011;**12**:634-44.

6 Ritchie ME, Diyagama D, Neilson J, van Laar R, Dobrovic A, Holloway A*, et al.* Empirical array quality weights in the analysis of microarray data. *BMC Bioinformatics* 2006;**7**:261.

7 Benjamini Y, Hochberg Y. Controlling the False Discovery Rate - a Practical and Powerful Approach to Multiple Testing. *Journal of the Royal Statistical Society Series B-Methodological* 1995;**57**:289-300.

8 Razick S, Magklaras G, Donaldson IM. iRefIndex: a consolidated protein interaction database with provenance. *BMC Bioinformatics* 2008;**9**:405.

9 Yoon J, Blumer A, Lee K. An algorithm for modularity analysis of directed and weighted biological networks based on edge-betweenness centrality. *Bioinformatics* 2006;**22**:3106-8.

10 Jostins L, Ripke S, Weersma RK, Duerr RH, McGovern DP, Hui KY*, et al.* Host-microbe interactions have shaped the genetic architecture of inflammatory bowel disease. *Nature* 2012;**491**:119-24.

11 Trynka G, Hunt KA, Bockett NA, Romanos J, Mistry V, Szperl A*, et al.* Dense genotyping identifies and localizes multiple common and rare variant association signals in celiac disease. *Nat Genet* 2011;**43**:1193-201.

12 Tsoi LC, Spain SL, Knight J, Ellinghaus E, Stuart PE, Capon F*, et al.* Identification of 15 new psoriasis susceptibility loci highlights the role of innate immunity. *Nat Genet* 2012;**44**:1341-8.

13 Chen EY, Xu H, Gordonov S, Lim MP, Perkins MH, Ma'ayan A. Expression2Kinases: mRNA profiling linked to multiple upstream regulatory layers. *Bioinformatics* 2012;**28**:105-11.

14 Johnson AD, Handsaker RE, Pulit SL, Nizzari MM, O'Donnell CJ, de Bakker PI. SNAP: a web-based tool for identification and annotation of proxy SNPs using HapMap. *Bioinformatics* 2008;**24**:2938-9.

15 Boyle AP, Hong EL, Hariharan M, Cheng Y, Schaub MA, Kasowski M*, et al.* Annotation of functional variation in personal genomes using RegulomeDB. *Genome Res* 2012;**22**:1790-7.
