# Supplementary material for: Generation of primary human intestinal T cell transcriptomes reveals differential expression at genetic risk loci for immune-mediated disease
Source: Gut. 2014 May 5;64(2):250–9. doi: 10.1136/gutjnl-2013-306657 (PMC4316924; doi:10.1136/gutjnl-2013-306657)
Supplement: Web figures [file gutjnl-2013-306657-s2.pdf]

Supplemental Figure 1 Sample acquisition and processing to ensure high data quality. **(a-f)** $CD4^{+}$ and $CD8^{+}$ T cell effector memory subpopulations were selected by polychromatic flow sorting. Flow cytometry was used to define cells as T cells if they fell within a lymphocyte gate (defined on forward-scatter/sidescatter), a singlet gate (defined on forward-scatter height vs. width), and a series of polychromatic gates defined as $CD3^{+}$ $TCR\alpha\beta^{+}$ $CD19^{-}$ $CD326^{-/lo}$ $DAPI^{-}$. Staining for CD62L and CD45RO were used to indicate cellular activation and memory status, with T_{EM} status defined as $CD45RO^{+}$ $CD62L^{-}$. The same approach was applied to LPLs **(a,b)**, IELs **(c,d)** and peripheral blood cells **(e,f)**. T cells were then further subcharacterised as $CD4^{+}$ $CD8\alpha^{-}$ $CD8\beta^{-}$ ($CD4^{+}$, **a,c,e**) or $CD4^{-}$ $CD8\alpha^{+}$ $CD8\beta^{+}$ ($CD8^{+}$, **b,d,f**). The percentage of the parent population falling within each quadrant is shown in red. T_{EM} subpopulations defined in this way were sorted for RNA extraction. Plots are representative examples from a single subject. **(g)** RNA degradation plot for each of the 36 arrays. Individual probes in each probeset are ordered by location relative to the 5' end of the targeted RNA molecule and scaled to uniform standard deviation. Average probe intensity at each location is plotted relative to a fixed 5' starting point that is incrementally shifted for each array. p values for testing the gradient of all slopes as non-zero were nonsignificant. **(h)** Boxplots representing summaries of the log_2 signal intensity distribution of the arrays after RMA processing and batch correction; computation of the Kolmogorov-Smirnov statistic K_a did not detect any array as an outlier.

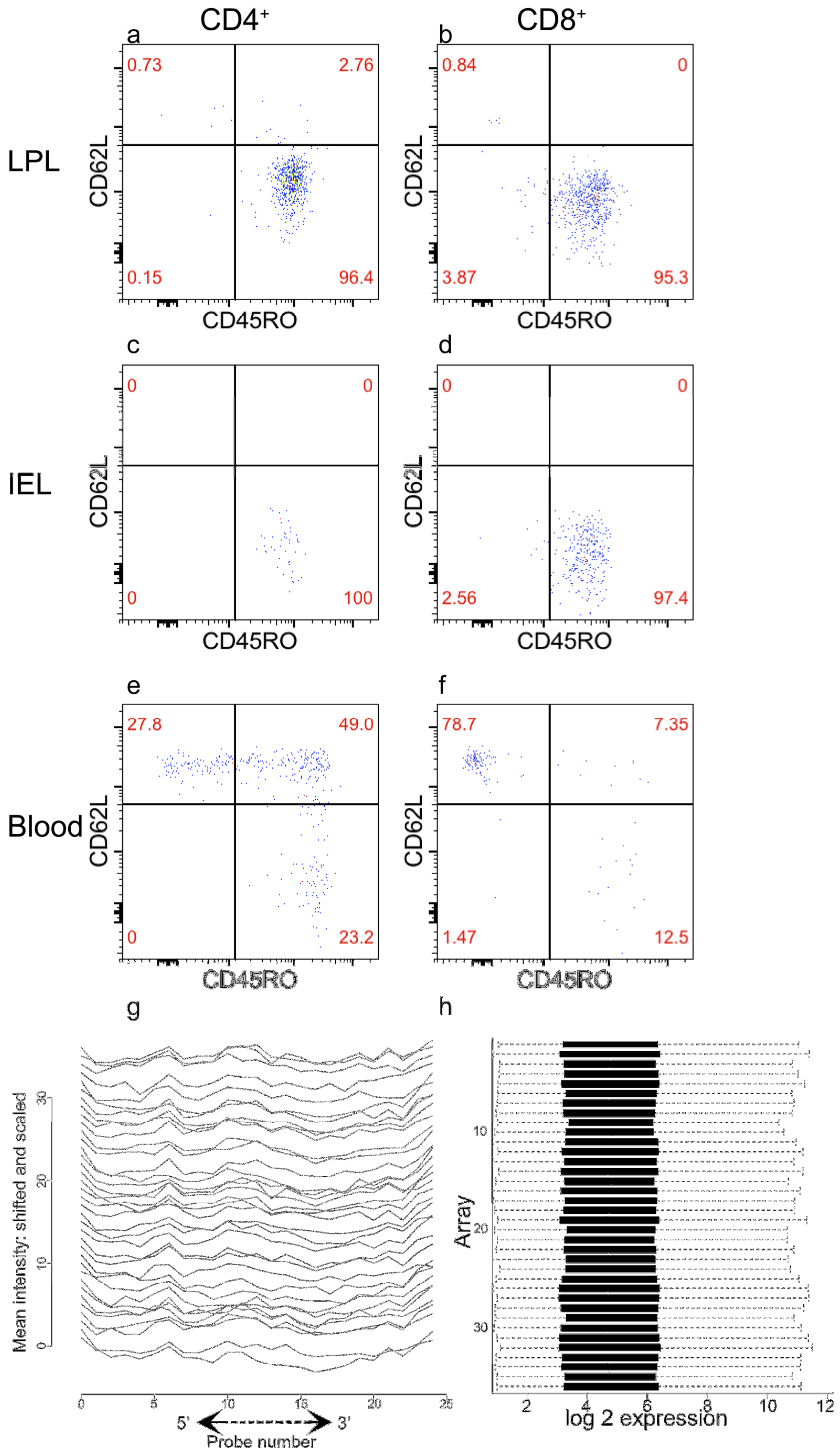

Raine *et al.* Supplemental Figure 1

Supplemental Figure 2 Gut T_{EM} cell populations show both shared and unique differentially expressed genes. **(a,b)** For each gut T_{EM} cell population, lists of genes showing upregulation (**a**) and downregulation (**b**) compared to paired peripheral blood T_{EM} cells were compared for shared and distinct members. Differential expression was defined as ≥1.4 fold change with p<0.05 after adjustment for multiple testing. Euler diagrams are shown with areas of overlap approximating to the proportion of overlap between lists, as well as absolute numbers of shared transcripts.

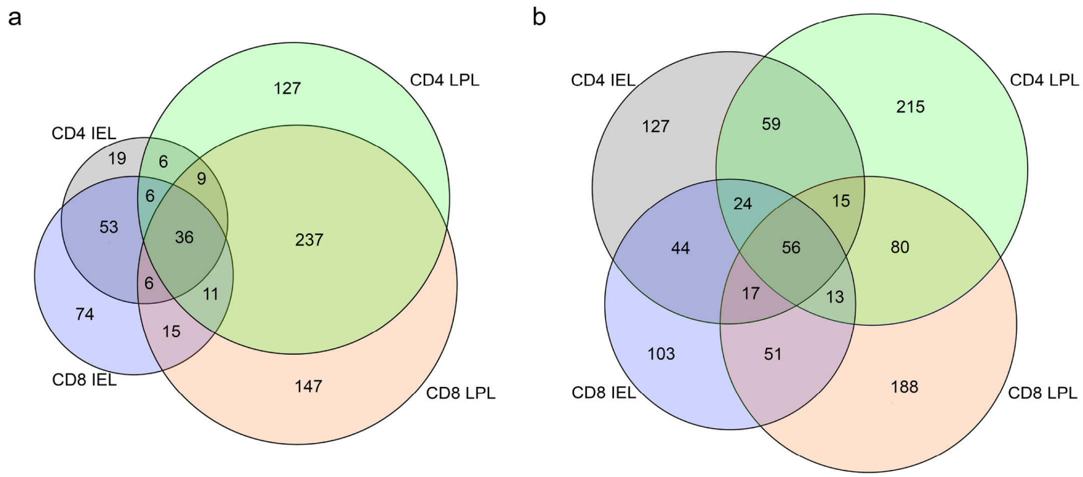

Raine *et al.* Supplemental Figure 2

Supplemental Figure 3 Gut T_{EM} subsets express a core subset of transcripts showing similar patterns of expression relative to peripheral blood T_{EM} cells. **(a,b)** Transcripts are listed that show upregulation (**a**) or downregulation (**b**) across all gut T_{EM} subsets relative to their paired peripheral blood T_{EM} cell counterparts. The fold-change is indicated, and the background shaded to reflect this, according to the key.

a

Gene	CD4 IEL	CD8 IEL	CD4 LPL	CD8 LPL
FOS	35.76	22.23	48.99	55.15
GPR34	21.85	48.88	6.54	19.70
FOSB	17.56	14.90	28.72	32.27
RGS1	15.55	10.91	34.04	31.31
RASGEF1B	2.29	2.19	34.01	34.41
CCL20	7.77	4.54	39.22	6.33
EGR1	13.89	10.18	13.76	16.17
IL2	5.99	7.00	17.18	14.54
JUN	9.72	12.58	8.71	11.88
CD69	6.40	6.68	12.89	12.87
PELO	7.59	13.06	8.02	10.12
SPINK2	6.11	13.85	4.33	11.97
ITGAE	10.75	11.45	3.15	5.96
SPRY1	3.61	4.41	8.75	12.98
LRRN3	4.68	8.91	4.22	9.19
CCR9	4.55	7.84	5.71	5.95
PPP1R15A	2.79	2.73	8.68	9.02
SKIL	3.76	3.89	7.63	6.47
CD9	4.22	7.12	1.92	4.49
CD101	4.85	6.07	2.50	3.02
ASB2	4.16	4.45	3.74	3.79
JUNB	2.73	2.30	4.55	4.20
EPAS1	2.41	2.74	3.68	4.75
DUSP6	3.98	3.37	2.80	2.84
NDFIP2	3.13	4.27	2.65	2.06
DSE	3.33	3.81	2.77	2.07
FURIN	2.87	1.89	4.44	2.55
ARHGAP18	2.85	3.95	2.11	2.79
CA10	3.08	3.31	2.33	2.26
ABCB1	3.57	2.69	1.99	2.13
ARHGEF12	2.40	2.27	2.75	2.23
C18orf1	1.74	2.17	2.56	2.99
TMEM200A	2.41	3.17	1.70	1.96
MAN1A1	2.63	1.88	2.03	1.77
ARHGAP9	2.01	1.78	2.06	2.04
LAG3	1.91	1.54	2.42	1.57

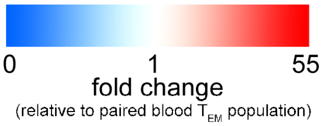

fold change
(relative to paired blood T_{EM} population)

b

Gene	CD4 IEL	CD8 IEL	CD4 LPL	CD8 LPL
CX3CR1	0.07	0.02	0.14	0.04
S1PR1	0.06	0.05	0.13	0.10
SAMD3	0.08	0.08	0.13	0.09
RASA3	0.08	0.06	0.19	0.19
CFH	0.12	0.12	0.14	0.17
PLEK	0.14	0.08	0.25	0.11
KLRG1	0.06	0.03	0.38	0.12
LEF1	0.08	0.08	0.27	0.27
PXN	0.19	0.13	0.26	0.18
KLRF1	0.22	0.05	0.40	0.09
GZMH	0.17	0.14	0.29	0.21
ITGB1	0.18	0.23	0.17	0.26
FAM65B	0.13	0.16	0.27	0.29
C1orf162	0.14	0.26	0.21	0.27
VCL	0.25	0.21	0.24	0.21
KLF3	0.15	0.11	0.38	0.30
GNLY	0.14	0.21	0.23	0.42
ITGA6	0.23	0.23	0.28	0.27
CDC25B	0.22	0.28	0.24	0.29
C1orf38	0.25	0.23	0.34	0.21
CTSW	0.22	0.27	0.27	0.29
LY75	0.31	0.32	0.36	0.26
C12orf75	0.21	0.31	0.35	0.37
AGTPBP1	0.20	0.29	0.43	0.33
TMEM55A	0.24	0.33	0.31	0.41
ZNF683	0.32	0.27	0.40	0.32
STK38	0.31	0.33	0.34	0.34
FLNA	0.25	0.35	0.35	0.44
HLA-DPB1	0.28	0.30	0.40	0.42
ADD3	0.35	0.41	0.38	0.30
ITGB2	0.35	0.23	0.53	0.33
DPH5	0.35	0.44	0.32	0.39
ST6GAL1	0.23	0.33	0.46	0.49
TMEM63A	0.40	0.31	0.41	0.41
S1PR4	0.38	0.40	0.38	0.39
D4S234E	0.34	0.36	0.37	0.48
NHSL2	0.28	0.37	0.45	0.51
ARRB1	0.47	0.37	0.48	0.31
NOSIP	0.40	0.46	0.40	0.40
SH3BP5	0.42	0.44	0.41	0.42
EOMES	0.49	0.27	0.52	0.41
TXNIP	0.52	0.56	0.32	0.30
CAPN2	0.33	0.51	0.33	0.54
IQSEC1	0.36	0.40	0.46	0.49
PLSCR3	0.34	0.54	0.34	0.53
GPX7	0.42	0.49	0.57	0.40
IL10RA	0.37	0.46	0.51	0.54
PDZD4	0.40	0.47	0.44	0.61
RAP2B	0.42	0.45	0.50	0.58
BIN2	0.38	0.54	0.52	0.52
NKG7	0.41	0.65	0.37	0.56
PIK3R5	0.41	0.43	0.64	0.55
ARHGAP25	0.52	0.55	0.49	0.54
GTF3A	0.52	0.61	0.54	0.54
ARHGEF18	0.57	0.52	0.58	0.61
DPEP2	0.52	0.57	0.62	0.61

Raine *et al.* Supplemental Figure 3

Supplemental Figure 4 Protein-protein interaction networks reveal centrality of AP-1 signalling in gut T_{EM} cells. **(a-d)** Transcripts overexpressed in IEL $CD4^+$ T_{EM} cells relative to blood $CD4^+$ T_{EM} cells **(a)**, IEL $CD8^+$ T_{EM} cells relative to peripheral blood $CD8^+$ T_{EM} cells (**b**), LPL $CD4^+$ T_{EM} cells relative to peripheral blood $CD4^+$ T_{EM} cells (**c**), and LPL $CD8^+$ T_{EM} cells relative to peripheral blood $CD8^+$ T_{EM} cells (**d**) were used to seed a protein-protein interaction networks. All proteins showing evidence of direct interaction with the seed protein list were included in the networks, as described in the Methods. Nodes, representing individual transcripts are distributed based upon strength of interaction data, and both nodes and edges are given prominence based upon criticality to overall network structure, as described in the Methods.

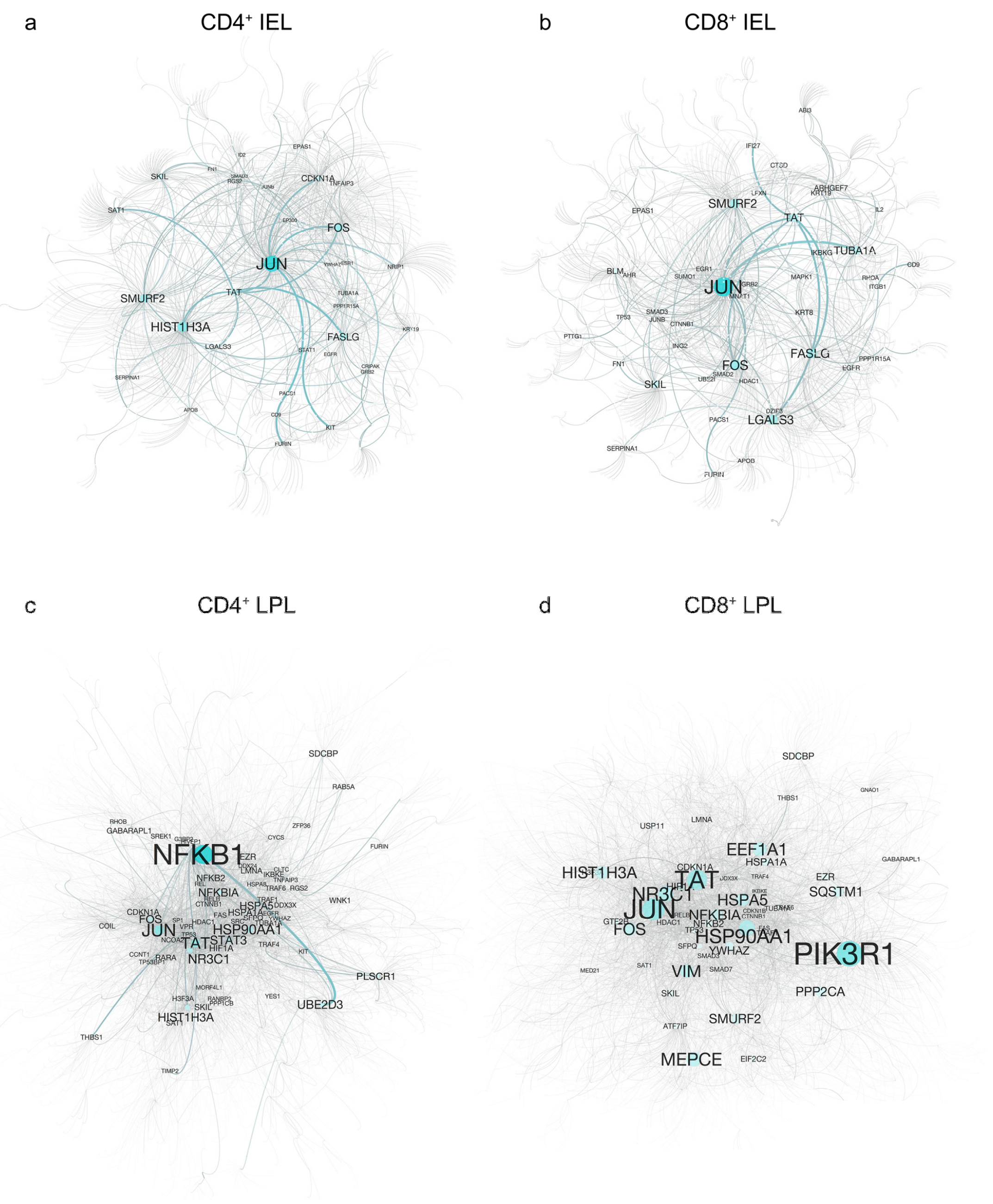

Raine *et al.* Supplemental Figure 4

Supplemental Figure 5 Risk loci for intestinal inflammatory pathologies are not enriched for genes upregulated in blood TEM populations relative to blood. Genetic risk loci associated with a range of diseases and traits, as indicated, were tested for overlap with transcripts showing downregulation in specific gut TEM populations relative to paired peripheral blood TEM populations, according to the algorithm illustrated in Figure 2 and as described in the methods. The proportion of intervals containing one or more downregulated genes within a window extending 0.2 cM either side of the lead SNP, is shown for each trait/gene list combination, with background colouring indicating the significance of the observation, as per the legend.

	Trait / Disease	Crohn's	UC	Coeliac	PBC	T1D	SLE	RhA	Psoriasis	Asthma	MS	BMI	Height	Cholesterol
	SNPs	140	133	31	23	39	24	32	29	19	51	37	188	52
Expression comparison	Subset													
Blood upregulated v LPL	CD4	0.14	0.14	0.10	0.13	0.10	0.17	0.13	0.14	0.16	0.20	0.08	0.13	0.12
	CD8	0.12	0.15	0.06	0.09	0.03	0.17	0.13	0.14	0.16	0.24	0.14	0.09	0.15
Blood upregulated v IEL	CD4	0.12	0.13	0.13	0.22	0.13	0.17	0.13	0.10	0.26	0.18	0.08	0.11	0.13
	CD8	0.14	0.12	0.13	0.13	0.13	0.17	0.13	0.07	0.21	0.20	0.11	0.10	0.15

$1x10^{-3}$

p value

$5x10^{-2}$

Raine *et al.* Supplemental Figure 5

Supplemental Figure 6 Differences in *ex vivo* cell handling do not stimulate differences in gene expression observed in CD4+ T_{EM} cells. Peripheral blood mononuclear cells isolated from healthy control individuals were subjected to conditions simulating the process used to extract IEL or LPL (or left 'untreated' on ice) prior to antibody labelling and cell sorting to isolate CD4+ T_{EM} cells. These were used for expression microarray analysis using exactly the same laboratory and computational pipeline as in the original manuscript. RMA normalised expression values (after filtering for detected coding transcripts as in the original manuscript) are shown compared to expression values from 'untreated' cells for cells exposed to EDTA/DTT (a) or cells exposed to EDTA/DTT then collagenase digestion (b). Coefficients of determination (denoted as r^2) were determined foreach correlation as shown. Using the same criteria as in the main study to determine differentially expressed genes between these cell populations (adj. *p* value <0.05, fold-change≥ ⍰}1.4), only 5 transcripts showed differential expression between Blood-EDTA/DTT and untreated blood CD4+ T_{EM} cells, and only 1 transcript between Blood-EDTA/DTT-collagenase and untreated blood CD4+ T_{EM} cells.

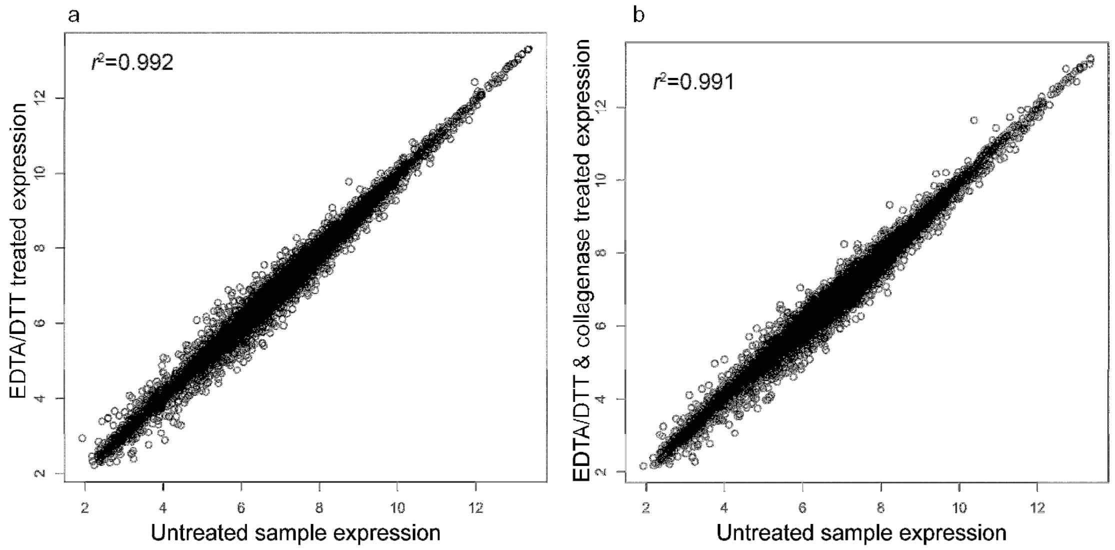

Supplemental Table 1 Transcription factor enrichment analysis suggests HNF4a as a key regulator of LPL T_{EM} genes with binding sites modified by IBD associated SNPs. A database of human ChIP-Seq data was interrogated to find transcription factors predicted to regulate genes upregulated in the LPL $CD4^+$ and $CD8^+$ T_{EM} cell populations (relative to paired peripheral blood T_{EM} cell populations). A *p* value for each transcription factor was calculated based upon the frequency of ChIP-Seq targets within the lists of differentially expressed genes, compared to the total frequency of targets within the total background ChIP-Seq dataset, and corrected for multiple testing. Next, all focal SNPs reported for IBD, as well as all SNPs in tight linkage disequilibrium, were analysed for the presence of binding sites for the transcription factors identified as potentially active, using a different expert curated ChIP-Seq database. Finally, for those transcription factors showing multiple potential binding sites modified by IBD SNPs (and SNPs in linkage disequilibrium), we used a Chi-squared test to assess the distribution of binding sites between those IBD-associated SNPs that overlapped a gene upregulated in the relevant gut T_{EM} population, and those IBD-associated SNPs that did not show such overlap. In this way, for both $CD4^+$ and $CD8^+$ LPL T_{EM} cells, HNF4a emerged as a potentially active transcription factor, with a significant enrichment of binding sites modified by IBD-associated SNPs whose genetic risk loci contained an upregulated gene.

	**TF prediction**				**SNP-TF scoring**		
Gene list	*Transcription factor*	*Fraction of targets in diff gene list*	*Fraction of targets in database*	*adjusted p value*	*targets / overlap SNPs*	*targets / non-overlapped SNPs*	*p value*
**$CD4^+$ LPL↑**	GATA2	0.2104	0.0803	1.11E-16	5/29	16/134	0.4398
	HNF4A	0.3522	0.2027	1.05E-12	9/29	16/134	0.0097
**$CD8^+$ LPL↑**	GATA2	0.2080	0.0803	3.61E-17	7/34	14/129	0.1317
	HNF4A	0.3562	0.2027	4.84E-14	10/34	15/129	0.0105
	CDX2	0.0420	0.0168	3.53E-04	6/34	16/129	0.4260
	SPI1	0.0730	0.0416	1.67E-03	5/34	15/129	0.6265

Raine *et al.* Supplemental Table 1
